# Methylxanthines Induce a Change in the AD/Neurodegeneration-Linked Lipid Profile in Neuroblastoma Cells

**DOI:** 10.3390/ijms23042295

**Published:** 2022-02-18

**Authors:** Daniel Janitschke, Anna Andrea Lauer, Cornel Manuel Bachmann, Jakob Winkler, Lea Victoria Griebsch, Sabrina Melanie Pilz, Elena Leoni Theiss, Heike Sabine Grimm, Tobias Hartmann, Marcus Otto Walter Grimm

**Affiliations:** 1 Experimental Neurology, Saarland University, 66421 Homburg, Germany; daniel.janitschke@uks.eu (D.J.); anna.lauer@uks.eu (A.A.L.); manuel.bachmann@uks.eu (C.M.B.); jakob.winkler@uks.eu (J.W.); lea@ifuws.de (L.V.G.); pilz.sabrina1995@gmail.com (S.M.P.); elena.theiss@web.de (E.L.T.); heike.grimm@gmx.de (H.S.G.); tobias.hartmann@uks.eu (T.H.); 2 Deutsches Institut für Demenzprävention, Saarland University, 66421 Homburg, Germany; 3 Nutrition Therapy and Counseling, Campus Rheinland, SRH University of Applied Health Science, 51377 Leverkusen, Germany

**Keywords:** lipidomics, methylxanthines, caffeine, theobromine, theophylline, pentoxifylline, propentofylline, sphingomyelin, ceramide, phosphatidylcholine, plasmalogens

## Abstract

Alzheimer’s disease (AD) is characterized by an increased plaque burden and tangle accumulation in the brain accompanied by extensive lipid alterations. Methylxanthines (MTXs) are alkaloids frequently consumed by dietary intake known to interfere with the molecular mechanisms leading to AD. Besides the fact that MTX consumption is associated with changes in triglycerides and cholesterol in serum and liver, little is known about the effect of MTXs on other lipid classes, which raises the question of whether MTX can alter lipids in a way that may be relevant in AD. Here we have analyzed naturally occurring MTXs caffeine, theobromine, theophylline, and the synthetic MTXs pentoxifylline and propentofylline also used as drugs in different neuroblastoma cell lines. Our results show that lipid alterations are not limited to triglycerides and cholesterol in the liver and serum, but also include changes in sphingomyelins, ceramides, phosphatidylcholine, and plasmalogens in neuroblastoma cells. These changes comprise alterations known to be beneficial, but also adverse effects regarding AD were observed. Our results give an additional perspective of the complex link between MTX and AD, and suggest combining MTX with a lipid-altering diet compensating the adverse effects of MTX rather than using MTX alone to prevent or treat AD.

## 1. Introduction

**Figure 1:**
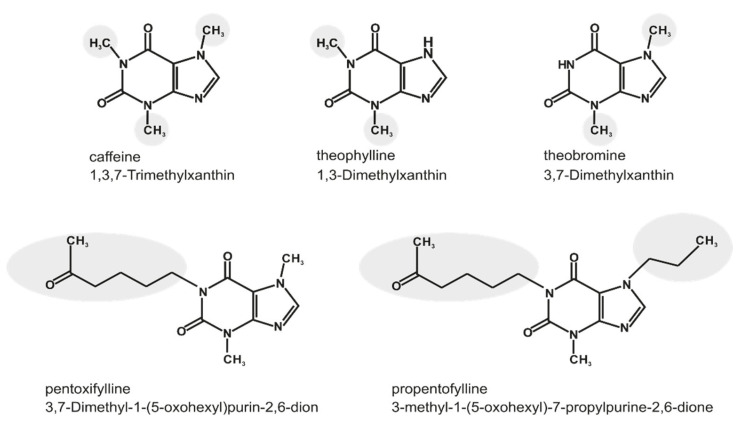
Chemical structure of xanthine and side chain combinations of its derivatives caffeine, theophylline, theobromine, pentoxifylline and propentofylline. Structural changes between the different methylxanthines are highlighted.

Methylxanthines (MTXs) are derivatives of the purine base xanthine and plant alkaloids, present in frequently consumed foods and beverages like coffee, cacao, chocolate and tea [1]. They are quickly absorbed in the gastrointestinal tract and distributed via the blood to different tissues, amongst others into the brain by crossing the blood–brain barrier [2]. Besides their well-known psychostimulant properties, MTXs show several neuroprotective aspects, e.g., decreased oxidative stress [3] and modulation of the inflammatory immune response by inhibition of pro-inflammatory mediator release and attenuation of microglia cell activation [4]. On the molecular level, MTXs antagonize cerebral adenosine receptors/purinergic P1 receptors because of their structural similarity with purine [5]. Four subtypes of these receptors are reported (A1, A2A, A2B and A3), which are expressed in different cells of the human body [6,7]. Further functions of MTXs, especially in higher dosage, are intracellular calcium mobilization, phosphodiesterase inhibition, and modulation of GABA_A_ receptors [8,9,10]. MTXs have therefore not only beneficial effects in respect to neurodegenerative diseases like schizophrenia, Parkinson’s disease and Alzheimer’s disease (AD) [11,12,13,14], but are also discussed in the prevention of several diseases, including respiratory tract and cardiovascular diseases [15,16,17]. In the present study, the following MTXs were used: caffeine, theophylline, and theobromine as naturally occurring, as well as pentoxifylline and propentofylline as synthetic MTXs. They differ in their structure in regard to the position and kind of their side chain, as shown in Figure 1. Theobromine is besides caffeine the most abundant MTX in cacao [18]. This molecule is characterized by two methyl groups at R2 and R3 at the nitrogens of the xanthine structure (Figure 1), and antioxidant properties have been reported [19]. Additionally, theobromine is discussed to have beneficial effects on cardiovascular dysfunction and respiratory diseases, but as these have to be validated, it is currently not used as medicinal drug. Theobromine does not have those strong stimulating properties on the central nervous system in comparison to caffeine. This could be explained by its lower adenosine receptor binding affinity, and additionally by the fact that theobromine has no metabolites like the trimethylxanthine caffeine, which can be metabolized to the dimethylxanthine theophylline having two methyl groups at R1 and R2 (Figure 1) [20,21]. This molecule is described to have a higher affinity to the adenosine receptors than caffeine [22]. Since theophylline also has bronchodilating properties, it has been the most widely prescribed xanthine used in the treatment of respiratory diseases, like asthma or chronic obstructive pulmonary disease (COPD). However, because of its side effects and increased effectiveness of other inhibitors, theophylline has become a third-line therapy [23,24]. Pharmaceutically-used synthetic MTXs are characterized by further functional groups at R1 and R2 beside the methyl group at the R2 position. The synthetic MTX pentoxifylline with two methyl groups at R2 and R3 additionally has an oxohexyl group at the R1 position. Because of its hemorrheologic and vasodilatation properties, this drug is used in the treatment of intermittent claudication [25]. Furthermore, some cardiac effects like elevated heart rate, cardiac output and systemic pressure are reported for pentoxifylline in dogs [26]. The second analyzed synthetic MTX is propentofylline (PPF), characterized by an oxohexyl group at the R1 position and a propyl group at position R3 in addition to the methylgroup at R2 (Figure 1). Neuroprotective, anti-proliferative and anti-inflammatory properties are described for propentofylline, and clinically it has demonstrated potency in neurodegenerative diseases, like dementia or multiple sclerosis, and moreover in schizophrenia [27]. Furthermore, it is a glial modulating agent with preventative effects regarding mechanical allodynia [28]. Several studies reported an influence of methylated xanthine derivatives on the regulation of the lipid homeostasis [29,30,31]. Furthermore, extensive lipid alterations have been reported in AD post-mortem brains and are discussed to be linked to the pathogenesis of AD. In our study we used a high-throughput lipidomic profiling technique to examine the effects of caffeine, theophylline, pentoxifylline, theobromine and propentofylline on the lipid homeostasis in human and murine neuroblastoma cells (SH-SY5Y and N2a). The aim of our study was to highlight which of the over 180 analyzed lipid species, including phosphatidylcholine (PC aa), lyso-phosphatidylcholine (Lyso-PC), phosphatidylcholine-plasmalogens (PC ae), triacylglyceride (TAG), sphingomyelin (SM), ceramide (Cer) and cholesterol are affected by a long-term incubation with the above-mentioned MTXs, to get detailed insights into potential beneficial properties of the different MTXs in respect to lipid alterations observed in AD.

## 2. Results

To analyze if methylated xanthine derivatives have an impact on lipid homeostasis in neuronal cells, we treated human (SH-SY5Y) and murine (N2a) neuroblastoma cells with the natural occurring MTXs caffeine, theobromine, theophylline and the synthetic ones pentoxifylline and propentofylline. In a semi-quantitatively shotgun mass spectrometry (MS) analysis, 188 different lipids including phospholipids (phosphatidylcholine (PC aa), lyso-phosphatidylcholine (Lyso-PC), phosphatidylcholine-plasmalogens (PC ae), sphingomyelin (SM)), neutral lipids (triglycerides (TAG)), cholesterol and ceramides (Cer) were measured. Deuterated standards of each lipid class were used to normalize the obtained data, which were further calculated as x-fold change compared to the cells treated with water as solvent control and moreover as mol%. The relative lipid changes in the examined species in MTX-treated cells compared to cells treated with the solvent control are shown in volcano plots and boxplots for each analyzed cell line. Throughout the complete manuscript, the volcano plots are divided in eight squares to represent (a) lipid parameters that showed a non-significant de- or increase smaller than the standard deviation (SD) in grey; (b) non-significant changes with a magnitude of effect greater than the SD as symbols under the horizontal line indicating a *p* value < 0.05, which was set as statistical significance; and (c) significantly de- or increased parameters with a magnitude of effect more than the SD in the upper left or right square. In the corresponding boxplots for each cell line, that are placed under the volcano plots, the changes relative to solvent control treated cells are presented.

### 2.1. Phosphatidylcholine Species (PC aa)

**Figure 2:**
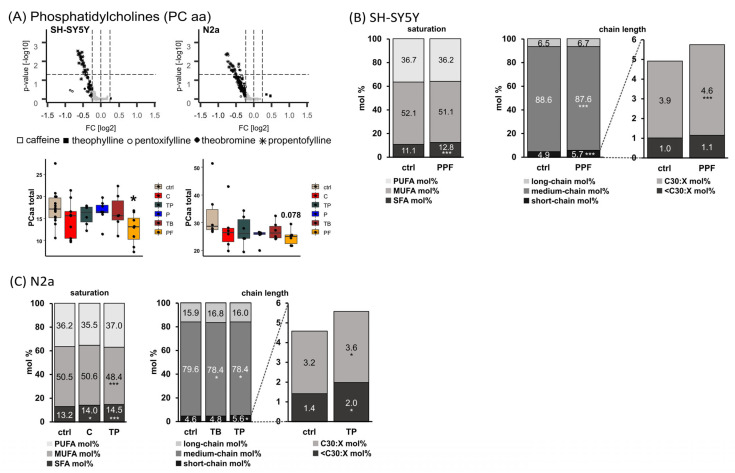
Alterations of phosphatidylcholine (PC aa) levels in different cell lines (SH-SY5Y and N2a) in the presence of the analyzed MTXs caffeine (C), theophylline (TP), pentoxifylline (P), theobromine (TB) and propentofylline (PPF). (**A**) PC aa total. Relative changes of PC aa species in comparison to cells treated with the solvent control are presented in volcano plots for each examined cell line. Lipid species with changes smaller than the standard deviation (SD) are marked in grey, while those changed greater than the SD are marked in black. *p*-values were calculated using Dunnett’s post-hoc test for each lipid class and statistical significance was set at *p* ≤ 0.05 (horizontal line in volcano plots). Below the volcano plots, the relative changes are presented in boxplots for the corresponding cell line as fold changes to the solvent control. (**B**) SH-SY5Y cells. Distribution of saturated (SFA), monounsaturated (MUFA) and polyunsaturated (PUFA), as well as short-, medium- and long-chain PC aa species in SH-SY5Y cells treated with PPF. Short-chain was defined as < C32:X, medium-chain as C32:X–C36:X and long-chain as > C36:X for PC aa throughout the manuscript. (**C**) N2a cells. Distribution of SFA, MUFA and PUFA as well as short-, medium- and long-chain PC aa species in N2a cells treated with C, TP, and TB. (**B**,**C**) are shown as boxplots in Supplemental Figure S2. (* *p* ≤ 0.05, *** *p* ≤ 0.001).

Incubation of MTXs led to a decrease of total PC aa species in the neuronal cell lines SH-SY5Y and N2a, as shown in the volcano plots and the boxplots (Figure 2A). The synthetic xanthine derivative propentofylline (PPF) reduced the total level of PC aa significantly to 72.1% (*p* = 0.018) in SH-SY5Y and to 74.5% (*p* = 0.078) in N2a cells, respectively.

Comparing the levels of PC aa species containing saturated fatty acids (SFA PC aa) in propentofylline treated SH-SY5Y cells with those in control-treated cells, a significant increase was found from 11.1 mol% to 12.8 mol% (*p* ≤ 0.001). In line with this, the short-chain (<32:X) PC aa species were significantly increased, and especially PC aa 30:X species were elevated from 3.9 mol% to 4.6% (*p* ≤ 0.001) in SH-SY5Y cells treated with propentofylline (Figure 2B).

In N2a cells, the naturally occurring MTXs caffeine and theophylline (TP) led to a significant increase in SFA PC aa (13.2 mol% to 14.0 mol% (*p* = 0.033) or 14.5 mol% (*p* ≤ 0.001), respectively), accompanied by a decrease in monounsaturated fatty acid (MUFA) PC aa species for theophylline (TP: 50.5 mol% to 48.4 mol%, *p* ≤ 0.001) (Figure 2C). In line with the findings for human neuroblastoma cells (SH-SY5Y), short-chain PC aa species (<32:X) were significantly increased in theophylline treated N2a cells, with < C30:X and C30:X species being significantly elevated (<C30:X from 1.4 mol% to 2.0 mol% (*p* = 0.049); C30:X from 3.2 mol% to 3.6 mol% (*p* = 0.013)). Moreover, theobromine, as well as theophylline, treatment led to a decrease in medium-chain PC aa species (C32:X–C36:X) from 79.6 mol% to 78.4 mol% (*p* = 0.031 for TB and *p* = 0.030 for TP).

### 2.2. Phosphatidylcholine Plasmalogen Species (PC ae)

**Figure 3:**
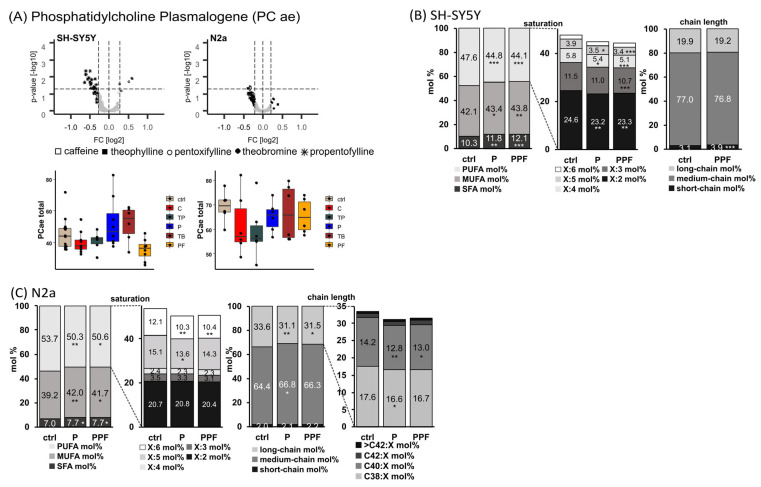
Alterations of phosphatidylcholine plasmalogen (PC ae) levels in different cell lines (SH-SY5Y and N2a) in the presence of the analyzed MTX caffeine (C), theophylline (TP), pentoxifylline (P), theobromine (TB) and propentofylline (PPF). (**A**) PC ae total. Relative changes of PC ae species in comparison to cells treated with the solvent control are presented in volcano plots for each examined cell line. Volcano plots are constructed as described in the caption of Figure 2. Below the volcano plots, the relative changes are presented in boxplots for the corresponding cell line as fold changes to the solvent control. (**B**) SH-SY5Y cells. Distribution of saturated (SFA), monounsaturated (MUFA) and polyunsaturated (PUFA), as well as short-, medium- and long-chain PC ae species in SH-SY5Y cells treated with P and PPF. Short-chain was defined as < C32:X, medium-chain as C32:X–C36:X and long-chain as > C36:X for PC ae throughout the manuscript. (**C**) N2a cells. Distribution of SFA, MUFA and PUFA, as well as short-, medium- and long-chain PC ae species in N2a cells treated with P and PPF. (**B**,**C**) are shown as boxplots in Supplemental Figure S3. (* *p* < 0.05, ** *p* < 0.01, *** *p* < 0.001).

Levels of total PC ae are not homogenously reduced in human and murine neuronal cell lines by MTX treatment like PC aa species, as described before. No significant MTX-induced alterations could be observed for PC ae total level in the analyzed cell lines, but pentoxifylline, as well as theobromine, tended to increase total PC ae levels in SH-SY5Y cells (Figure 3A). In respect to saturation of PC ae species in SH-SY5Y cells, the synthetic xanthine derivatives pentoxifylline and propentofylline significantly increased SFA from 10.3 mol% to 11.8 mol% (P, *p* = 0.002) and 12.1 mol% (PPF, *p* ≤ 0.001) and MUFA from 42.1 mol% to 43.4 mol% (P, *p* = 0.037) and 43.8 mol% (PPF, *p* = 0.004), respectively (Figure 3B). PUFA were significantly reduced from 47.6 mol% to 44.8 mol% (P, *p* ≤ 0.001) and 44.1 mol% (PPF, *p* ≤ 0.001) accordingly. Within the PUFA, all species containing two to five double bonds were significantly decreased, and those with six double bonds tended to decrease due to treatment with pentoxifylline or propentofylline. In line with the increase in SFA, propentofylline significantly elevated the levels of short-chain (< C32:X) PC ae species from 3.1 mol% to 3.9 mol% (*p* ≤ 0.001).

Similar results for pentoxifylline and propentofylline in respect to saturation of PC ae were found in murine neuroblastoma cells (Figure 3C). SFA species were significantly elevated from 7.0 mol% to 7.7 mol% by pentoxifylline (*p* = 0.042) and propentofylline (*p* = 0.049) and MUFA significantly increased (from 39.2 mol% to 42.0 mol% (P, *p* = 0.009) and 41.7 mol% (PPF, *p* = 0.018)). In line with the findings in SH-SY5Y cells, PUFA were reduced (from 53.7 mol% to 50.3 mol% (P, *p* = 0.009) and 50.6% (PPF, *p* = 0.016)). In particular, PUFA species containing five or six double bonds were decreased by the synthetic MTXs (X:5 from 15.1 mol% to 13.6 mol% (P, *p* = 0.011) and 14.3 mol% (PPF, not significant); X:6 from 12.1 mol% to 10.3 mol% (P, *p* = 0.001) and 10.4 mol% (PPF, *p* = 0.002)). In respect to chain length, pentoxifylline and propentofylline significantly increased medium-chain from 64.4 mol% to 66.8 mol% (*p* = 0.011) and 66.3 mol% (*p* = 0.052) and significantly decreased long-chain PC ae species from 33.6 mol% to 31.1 mol% (*p* = 0.007) and 31.5 mol% (*p* = 0.029), respectively. Within the long-chain species, C38:X and C40:X were significantly reduced in N2a cells due to the treatment with the synthetic MTXs.

To evaluate whether the analyzed xanthine derivatives have an impact on oxidative stress in the examined cell lines, the ratios between PC ae and PC aa total levels were calculated for each cell line after MTX incubation (Supplemental Figure S1). In SH-SY5Y and N2a cells, the PC ae total/PC aa total ratios tended to increase after incubation with pentoxifylline, theobromine and propentofylline. The ratio of PC ae SFA/PC aa SFA is significantly increased in SH-SY5Y cells due to pentoxifylline treatment (145.2%, *p* = 0.034). This is due to an increase in saturated PC ae species by pentoxifylline in these cell lines (see Figure 3). In N2a cells, the PC ae total/PC aa total ratio, as well as the PC ae SFA/PC aa SFA ratio and the PC ae MUFA/PC aa MUFA ratio, are significantly increased after pentoxifylline and propentofylline incubation (total ratios to 119.3% for P (*p* = 0.110) and to 122.6% for PPF (*p* = 0.047); SFA ratios to 131.2% for P (*p* = 0.010) and to 138.8 % for PPF (*p* = 0.001); MUFA ratios to 127.6% for P (*p* = 0.024) and to 129.4 % (*p* = 0.015)).

### 2.3. Lyso-Phosphatidylcholine Species (Lyso-PC)

**Figure 4:**
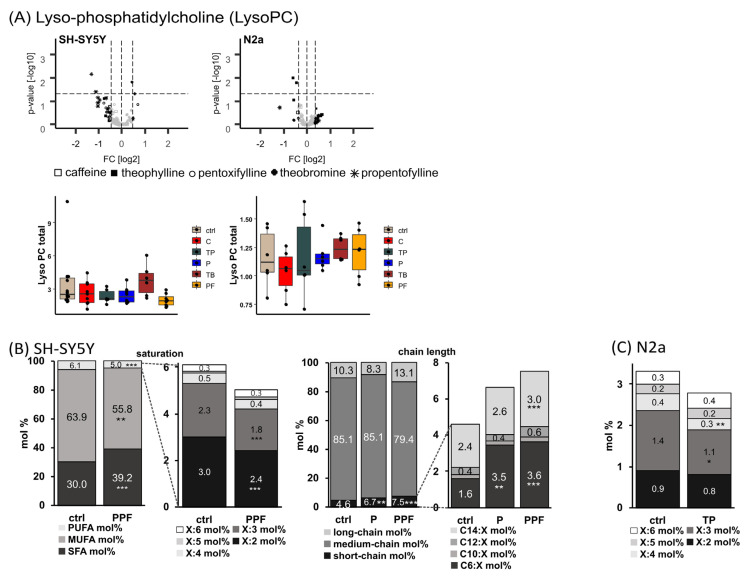
Alterations of lyso-phosphatidylcholine plasmalogen (Lyso-PC) levels in different cell lines (SH-SY5Y and N2a) in the presence of the analyzed MTX caffeine (C), theophylline (TP), pentoxifylline (P), theobromine (TB) and propentofylline (PPF). (**A**) Lyso-PC total. Relative changes of Lyso-PC species in comparison to cells treated with the solvent control are presented in volcano plots for each examined cell line. Volcano plots are constructed as described in the caption of Figure 2. Below the volcano plots, the relative changes are presented in boxplots for the corresponding cell line as fold changes to the solvent control. (**B**) SH-SY5Y cells. Distribution of saturated (SFA), monounsaturated (MUFA) and polyunsaturated (PUFA), as well as short-, medium- and long-chain PC ae species in SH-SY5Y cells treated with P and PPF. Short-chain was defined as < C16:X, medium-chain as C16:X–C20:X and long-chain as > C20:X for Lyso-PC species throughout the manuscript. (**C**) N2a cells. Distribution of PUFA Lyso-PC species in N2a cells treated with TP. (**B**,**C**) are shown as boxplots in Supplemental Figure S4. (* *p* < 0.05, ** *p* < 0.01, *** *p* < 0.001).

As a third phospholipid species, levels of Lyso-PC were analyzed in human and murine neuroblastoma cell lines after treatment with methylated xanthine derivatives (Figure 4). In SH-SY5Y cells, all analyzed MTXs except theobromine tended to decrease Lyso-PC levels to 58–77%, but without reaching statistical significance.

Evaluating the lipid changes in SH-SY5Y cells after MTX treatment in more detail, it became obvious that incubation with propentofylline led to significantly increased levels of Lyso-PC SFA from 30.0 mol% to 39.2 mol% (*p* ≤ 0.001) and significantly reduced levels of MUFA and PUFA Lyso-PC species from 63.9 mol% to 55.8 mol% (MUFA, *p* = 0.001) and from 6.1 mol% to 5.0 mol% (PUFA, *p* ≤ 0.001), respectively (Figure 4B). Within the Lyso-PC PUFA species, those with two or three double bonds are decreased significantly due to propentofylline treatment (X:2 from 3.0 mol% to 2.4 mol% (*p* ≤ 0.001); X:3 from 2.3 mol% to 1.8 mol% (*p* ≤ 0.001)). In respect to the chain length, pentoxifylline and propentofylline significantly elevated the levels of short-chain (< C16:X) Lyso-PC species from 4.6 mol% to 6.7 mol% (P, *p* = 0.008) and 7.5 mol% (PPF, *p* ≤ 0.001), respectively. In particular, C6:X and C14:X Lyso-PC species were increased (C6:X from 1.6 mol% to 3.5 mol% (P, *p* = 0.002) and 3.6 mol% (PPF, *p* = 0.001); C14:X from 2.4 mol% to 3.0 mol% (PPF, *p* = 0.001)). In line with the reduced PUFA Lyso-PC species found in SH-SY5Y cells, in the murine neuroblastoma cell line, N2a theophylline treatment tended to decrease Lyso-PC PUFA levels with significantly reduced levels of X:4 species from 0.4 mol% to 0.3 mol% (*p* = 0.001) (Figure 4C).

To examine if the analyzed MTXs have an influence on the activity of phospholipase A2 (PLA2), responsible for the cleavage of fatty acids in the sn-2 position of phospholipids, we calculated the ratio Lyso-PC/PC aa. Interestingly, the ratio of Lyso-PC SFA/PC aa SFA species was significantly increased in N2a cells due to treatment with pentoxifylline, theobromine and propentofylline (P to 133.9 % (p = 0.021); TB to 135.2 % (*p* = 0.016); PPF to 138.2 % (*p* = 0.008)) (Supplemental Figure S1). Similar results were obtained in SH-SY5Y cells: theobromine increased the ratio of Lyso PC SFA/PC aa SFA to 131.3% (*p* = 0.086) (Supplemental Figure S1).

### 2.4. Sphingomyelin (SM)

**Figure 5:**
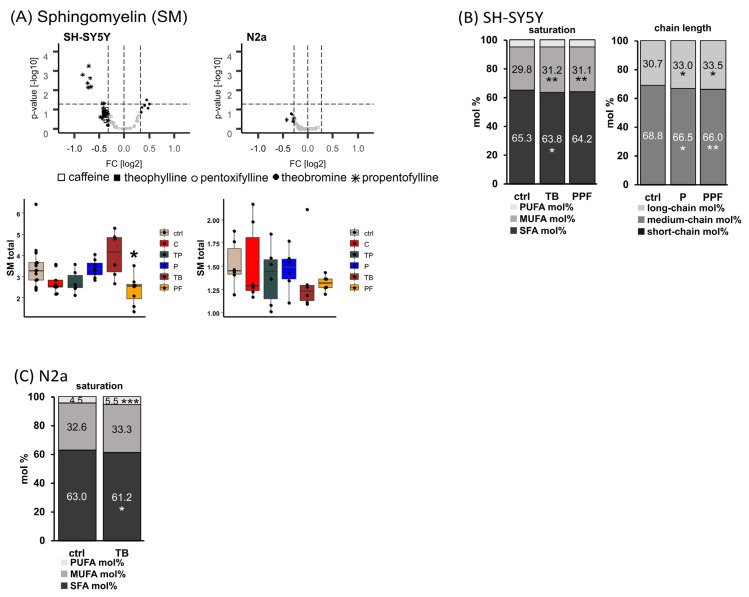
Alterations of sphingomyelin (SM) levels in different cell lines (SH-SY5Y and N2a) in the presence of the analyzed MTX caffeine (C), theophylline (TP), pentoxifylline (P), theobromine (TB) and propentofylline (PPF). (**A**) SM total. Relative changes of SM species in comparison to cells treated with the solvent control are presented in volcano plots for each examined cell line. Volcano plots are constructed as described in the caption of Figure 2. Below the volcano plots, the relative changes are presented in boxplots for the corresponding cell line as fold changes to the solvent control. (**B**) SH-SY5Y cells. Distribution of saturated (SFA), monounsaturated (MUFA) and polyunsaturated (PUFA), as well as short-, medium- and long-chain SM species in SH-SY5Y cells treated with TB, P and PPF. Short-chain was defined as <C32:X, medium-chain as C34:X–C38:X and long-chain as >C40:X for SM species throughout the manuscript. (**C**) N2a cells. Distribution of SFA, MUFA and PUFA SM species in N2a cells treated with TB. (**B**,**C**) are shown as boxplots in Supplemental Figure S5. (* *p* < 0.05, ** *p* < 0.01, *** *p* < 0.001).

Sphingomyelins are of great interest in respect to neurodegenerative diseases and were therefore included in our study. In Figure 5A, the total SM levels in neuronal cells of different origin after incubation with methylated xanthine derivatives are shown. The synthetic MTX propentofylline led to a significant reduction in SM total levels in the human neuroblastoma cell line SH-SY5Y to 68.8% (*p* = 0.016).

In SH-SY5Y cells, SFA SM species were significantly reduced from 65.3 mol% to 63.8 mol% (*p* = 0.029) due to treatment with theobromine, and MUFA SM species were significantly increased from 29.8 mol% to 31.2 mol% (*p* = 0005) by theobromine and to 31.1 mol% (0.006) by propentofylline (Figure 5B). In respect to the chain length, short-chain (<C32:X) SM were elevated from 0.5 mol% to 0.6 mol% (*p* = 0.003) by propentofylline, and medium-chain (C34:X–C38:X) were significantly decreased by the synthetic MTX pentoxifylline and propentofylline from 68.8 mol% to 66.5 mol% (P, *p* = 0.034) and to 66.0 mol% (PPF, *p* = 0.008). Long-chain (>C40:X) SM species were increased due to treatment with the synthetic MTX from 30.7 mol% to 33.0 mol% (P, *p* = 0.040) and to 33.5 mol% (PPF, *p* = 0.010).

In the murine neuroblastoma cell line, N2a treatment with theobromine resulted in significantly reduced SM SFA levels (from 63.0 mol% to 61.2 mol%, *p* = 0.040) accompanied by significantly elevated SM PUFA levels (from 4.5 mol% to 5.5 mol%, *p* = 0.001) (Figure 5C). The SM total/PC aa total ratio was increased to 123.2% (*p* = 0.081) when N2a cells were incubated with pentoxifylline (supplemental Figure S1).

To focus on anabolism and catabolism of sphingomyelins, we calculated the ratios SM total/PC aa total (anabolism) and Cer total/SM total (catabolism) for each cell line treated with the analyzed methylated xanthine derivatives. In SH-SY5Y cells, theobromine tended to increase SM anabolism to 124.2% (*p* = 0.057) (supplemental Figure S1). This trend became significant when comparing the ratios SM MUFA/PC aa MUFA and SM PUFA/PC aa PUFA of theobromine-treated cells with solvent control treated cells (MUFA: 127.7%, *p* = 0.030; PUFA: 130.6%, *p* = 0.042). In line with the finding that theobromine tended to increase the anabolism of SM species, the Cer total/SM total ratio tended to decrease in SH-SY5Y cells incubated with this naturally occurring methylxanthine to 63.7% without reaching statistical significance.

### 2.5. Ceramide (Cer)

Hydrolysis of sphingomyelin, which is catalyzed by sphingomyelinases, generates ceramide (Cer), a lipid molecule that is found within the cell membrane. Beside the generation of ceramides, which are a central part of the sphingolipid pathway, by sphingomyelin hydrolysis ceramide can be generated from sphingosine, ceramide-1-phosphate and 1-O-acylceramides, and can be synthesized de novo in the endoplasmic reticulum by a pathway that starts with the condensation of L-serine and palmitoyl CoA by the enzyme serine palmitoyl transferase.

**Figure 6:**
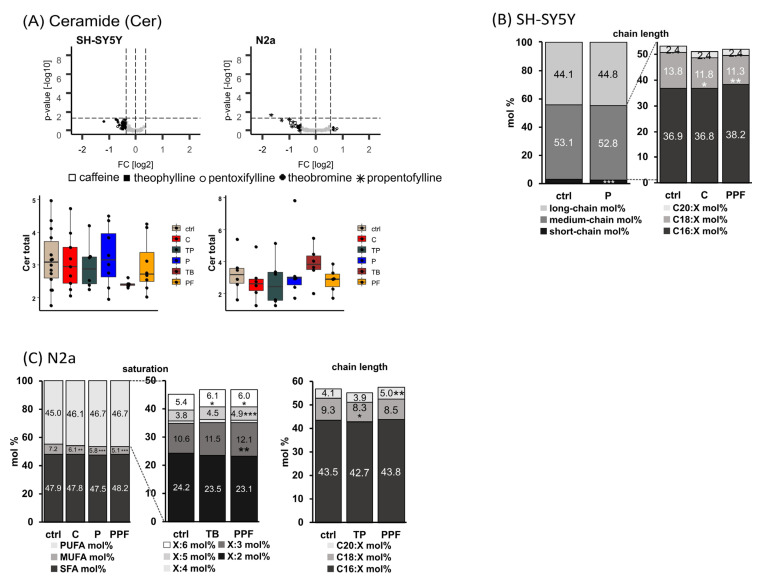
Alterations of ceramide (Cer) levels in different cell lines (SH-SY5Y and N2a) in the presence of the analyzed MTX caffeine (C), theophylline (TP), pentoxifylline (P), theobromine (TB) and propentofylline (PPF). (**A**) Cer total. Relative changes of Cer species in comparison to cells treated with the solvent control are presented in volcano plots for each examined cell line. Volcano plots are constructed as described in the caption of Figure 2. Below the volcano plots, the relative changes are presented in boxplots for the corresponding cell line as fold changes to the solvent control. (**B**) SH-SY5Y cells. Distribution of short-, medium- and long-chain Cer species in SH-SY5Y cells treated with P, C and PPF. Short-chain was defined as <C16:X, medium-chain as C16:X–C20:X and long-chain as >C20:X for Cer species throughout the manuscript. (**C**) N2a cells. Distribution of saturated (SFA), monounsaturated (MUFA) and polyunsaturated (PUFA), as well as short-, medium- and long-chain Cer species in N2a cells treated with C, P, TB, TP and PPF. (**B**,**C**) are shown as boxplots in Supplemental Figure S6. (* *p* < 0.05, ** *p* < 0.01, *** *p* < 0.001).

In SH-SY5Y cells, treatment with pentoxifylline led to a significant decrease in short-chain (<C16:X) Cer species from 2.8 mol% to 2.4 mol% (*p* = 0.001) (Figure 6B). Additionally, this MTX significantly increased the amount of C18:1 bound to the sn1 position from 91.8 mol% to 92.6 mol% by significantly decreasing that of C18:2 from 8.2 mol% to 7.4 mol% (*p* = 0.039) simultaneously. Within the medium-chain (C16:X–C20:X) Cer, caffeine and propentofylline significantly decreased the levels of C18:X from 13.8 mol% to 11.8 mol% (C, *p* = 0.037) and 11.3 mol% (PPF, *p* = 0.008).

Treatment with the analyzed xanthine derivatives decreased the levels of MUFA Cer species in N2a cells homogenously, with caffeine, pentoxifylline and propentofylline led to a significant reduction from 7.2 mol% to 6.1 mol% (C, *p* = 0.003), 5.8 mol% (*p*, *p* ≤ 0.001) and 5.1 mol% (PPF, *p* ≤ 0.001) (Figure 6C). Within the PUFA Cer species, propentofylline significantly elevated the levels of X:3, X:5 and X:6 species. In line, the naturally occurring MTX theobromine also increased the levels of X:6 significantly from 5.4 mol% to 6.1 mol% (*p* = 0.027). In respect to the chain length, theophylline significantly decreased C18:X Cer species from 9.3 mol% to 8.3 mol% (*p* = 0.040), and propentofylline significantly increased C20:X Cer species from 4.1 mol% to 5.0 mol% (*p* = 0.004).

### 2.6. Effect of Methylxanthines on Triaclyglycerides (TAG) and Cholesterol

Moreover, we included TAG and cholesterol in our lipidomics study. Incubation of the analyzed cell lines with the MTXs caffeine, theobromine, theophylline, pentoxifylline and propentofylline led to decreased levels of TAG species and cholesterol (Supplemental Figure S7).

## 3. Discussion

Lipids and lipid alterations are discussed to be important players in the development of neurodegenerative diseases, including Alzheimer’s and Parkinson’s disease [32,33]. Extensive lipid changes have been found in brains of AD-affected individuals and AD animal models. These alterations include, e.g., the level of total phospholipids, changes in phosphatidylcholine and phosphatidylethanolamine, plasmalogens, sphingomyelin, ceramide, cholesterol and DHA [33,34]. On the other hand, the release of the amyloid-β (Aβ) peptide from the amyloid precursor protein (APP) is strongly affected by lipids, and lipids have also been found to affect Aβ degradation [35,36,37,38,39,40]. These changes in Aβ production and degradation caused by specific lipids strongly affect the total level of Aβ peptides that aggregate in senile plaques of AD patients, one important pathological hallmark of the disease [41]. Recently, we and others have found that MTXs, that are frequently consumed in almost every area of the world, decrease Aβ generation and Aβ aggregation [13,42], and MTXs have been reported to display health benefits in many neurodegenerative diseases involving cell death in the nervous system [12]. Therefore, we analyzed in our present study whether MTXs also exert potential beneficial effects on lipids found to be changed in AD.

All analyzed MTXs tended to decrease the total PC aa level in both neuronal cell lines with the strongest effect found for the synthetic MTX propentofylline. In human neuroblastoma SH-SY5Y cells, propentofylline significantly decreased total PC aa to 72.1%, in murine N2a cells to 74.5% (*p* = 0.078). Interestingly, PC aa species containing saturated fatty acids were significantly increased by MTXs in neuronal cells (SH-SY5Y: PPF, N2a: C and TP). We found that MTXs significantly increased short-chain PC aa species (C30:X) in SH-SY5Y and N2a cells accompanied by a significant decrease in medium-chain PC aa species (C32-36:X). Incubation with MTXs also consistently reduced the level of TAG and cholesterol in these cell lines. The MTX-induced reduction of TAG and cholesterol might not only be beneficial in respect to AD where high cholesterol levels are reported to be closely linked to the disease [43,44], but also in respect to cardiovascular diseases, diabetes and metabolic syndrome. Taking into account that TAGs are stored in specialized cellular organelles, called lipid droplets, whose membranes are built of phospholipids, especially PC aa, changes in TAG level might also have an impact on PC aa. In line with this, we found an MTX-induced reduction of both total PC aa level and TAGs, for both neuronal cell lines.

In line with our findings that MTXs elevate SFA PC aa species, plasmalogen species (PC ae) containing saturated fatty acids (SFA) were significantly increased in the analyzed cell lines accompanied by a significant reduction of PUFA PC ae species. Interestingly, in both neuronal cell lines, SH-SY5Y and N2a, the effect on SFA PC ae and PUFA PC ae species was mediated by the synthetic MTXs pentoxifylline and propentofylline. These MTXs also significantly increased MUFA PC ae species. Consistently, for both neuronal cell lines, pentoxifylline and propentofylline reduced PC ae species with five and six double bonds that might include the omega-3 FAs eicosapentaenoic acid (EPA, C20:5) and DHA, which are reported to be decreased in brains of AD patients [45,46] and are closely linked with AD pathology [36,47,48,49]. These results indicate that propentofylline, as well as pentoxifylline, might aggravate the decline of EPA and DHA in brains of AD-affected individuals.

As plasmalogens are more susceptible for oxidation because of their enol ether bondage in the sn-1 position of the glycerol backbone compared to the corresponding ester-bonded glycerophospholipid, the ratio of PC ae/PC aa species represents a valuable indicator for oxidative stress. The synthetic MTXs pentoxifylline and propentofylline, as well as naturally occurring theobromine, tended to increase the ratio of PC ae total/PC aa total in both neuronal cell lines, indicating that MTXs might be useful in respect to ROS-associated diseases, including not only AD but also cancer, insulin resistance, diabetes mellitus, cardiovascular diseases, atherosclerosis, and aging in general. This finding is further substantiated by the significant increase in PC ae tot/PC aa tot, PC ae SFA/PC aa SFA and PC ae MUFA/PC aa MUFA in the murine neuronal cell line observed after treatment with pentoxifylline and propentofylline. Pentoxifylline also significantly elevated the ratio of PC ae SFA/PC aa SFA in the human neuroblastoma cell line. This synthetic MTX also revealed, besides theobromine, a trend to elevate total plasmalogen levels. These findings are of importance as plasmalogen levels are found to be decreased in the brain and blood of patients affected by AD [50,51,52,53]. In addition, intraperitoneal administration of plasmalogens improved cognitive function in an AD animal model [54], and oral administration of scallop-derived purified plasmalogens may improve cognitive functions of patients with mild AD [55]. The potential mechanisms underlying the beneficial effects of plasmalogens in respect to AD may be attributed to a plasmalogen-induced reduction in γ-secretase activity leading to decreased amyloid-β level [38] and/or reduced neuronal cell death, as plasmalogens have been reported to inhibit caspase-9 and caspase-3 cleavages in primary mouse hippocampal neuronal cells and to enhance phosphorylation of AKT and ERK signaling through the activation of orphan G-protein coupled receptor proteins [56]. Thus, the potency of MTX to increase plasmalogens or PC ae/PC aa ratios might be beside the known MTX-induced reduction in total Aβ-level [13], a further beneficial property in respect to AD.

Notably, a significant increase in SFA Lyso-PC species accompanied by a significant decrease in PUFA Lyso-PC species was found in SH-SY5Y cells in the presence of propentofylline. In human neuroblastoma cells, all analyzed MTX (except theobromine) revealed a trend to decrease total Lyso-PC level. An elevated activity of the phospholipase A2 (PLA2) primarily releasing arachidonic acid from the sn-2 position has been reported in brains of AD-affected individuals and in an AD mouse model [57,58]. Genetic ablation or reduction of PLA2 activity ameliorated cognitive deficits in this AD mouse model [58]. Thus, the observed MTX-induced reduction in total Lyso-PC level might indicate a reduced PLA2 activity in the presence of MTXs, which might be favorable in respect to AD. However, the impact of Lyso-PC regarding AD is controversially discussed in literature as Lyso-PC levels have also been found to be decreased in AD brains [59,60,61,62], which might be explained by the fact that Lyso-PC species are on the one hand produced by PLA2 activity, but represent, on the other hand, an important transporter of fatty acids in the brain. An impaired transport would consequently decrease Lyso-PC level in the brain.

The potential positive properties of MTXs in respect to AD are further underlined by changes in sphingomyelin (SM) and ceramide (Cer) level. By calculating the ratios of SM total/PC aa total, SM MUFA/PC aa MUFA and SM PUFA/PC aa PUFA, we found that theobromine increased SM anabolism in SH-SY5Y cells. The observed MTX-induced elevation of SM anabolism is further substantiated by our findings that theobromine in SH-SY5Y cells showed a strong trend to increase total SM level. As SM has been shown to decrease amyloid-β levels and SM levels have been found to be reduced in human brains and animal models of AD [63,64,65], the SM-increasing character of MTXs might be a further prospective property of MTX in respect to AD prevention or therapy. In line with this, theobromine significantly decreased (63.7%) the ratio of total ceramide/total sphingomyelin level (Cer total/SM total), indicating that theobromine also decreases the catabolism of SM to Cer. These changes in the ratio of Cer to SM might not only be relevant for AD, but also for depressive disorders. For AD, amyloid-β has been found to increase neutral sphingomyelinase (nSMase) activity, promoting the degradation of protective SM to Cer [63]. Similarly, an elevated acid sphingomyelinase (aSMase) activity has been reported to be involved in the pathophysiology of depressive disorders like major depression [66]. In general, the theobromine-induced decrease in SM catabolism decreasing Cer level might also display protection against apoptotic processes induced by Cer, not only relevant for AD but for many other diseases.

It is reasonable to assume that, at least in part, the observed effects of MTXs are mediated by the adenosine receptor/purinergic P1 receptor family. One might speculate that the different expression patterns of adenosine receptor A1 (*ADORA1*), adenosine receptor A2a (*ADORA2A*), adenosine receptor A2b (*ADORA2B*), and adenosine receptor A3 (*ADORA3*) detected in the analyzed cell lines (see Supplemental Figure S8) contribute to the differences observed in the lipid homeostasis upon MTX incubation. In human and murine neuroblastoma cell lines, *ADORA2A* showed the highest expression in the ADORA family, which is in line with findings that this adenosine receptor is highly expressed in brain [67,68] and neuroblastoma cells [69,70]. Interestingly, between human and murine neuroblastoma cells, differences in the *ADORA2B* and *ADORA3* expression pattern can also be observed. Whereas in N2a cells *ADORA2B* was not detectable under our experimental conditions, *ADORA3* was also slightly increased in SH-SY5Y cells compared to N2a. On the other hand, N2a revealed a higher expression of *ADORA2A* compared to SH-SY5Y cells. Nevertheless, further studies are needed to address the question of whether the observed alterations in the expression pattern of adenosine receptors in the analyzed cell lines might contribute to the MTX-induced changes in lipid homeostasis.

In summary, our results provide evidence that MTXs can induce lipid changes that might be beneficial in respect to AD and further diseases affected by lipid alterations. As discussed, these potential beneficial properties of MTXs include an elevation of plasmalogens and sphingomyelin, as well as a reduction of Lyso-PC. Beside these positive aspects, it must be mentioned that MTXs also exert potential negative properties: they reduce total PC aa level, known to be important for synaptogenesis [71,72], and they increase SFAs discussed to elevate the risk to develop AD [73,74,75]. Therefore, the potential application of MTXs in AD prevention or treatment implies a healthy diet with a high intake of long chain polyunsaturated fatty acids associated with a lower risk for AD and related dementias [36,75,76,77]. Furthermore, it must be mentioned that our analysis is a pure cell-culture study and that the effect of MTXs on lipid alterations has to be proven in vivo. However, the presented positive effects of MTXs on lipid alterations known to be affected in AD, together with the known MTX-induced reduction in total Aβ level and Aβ aggregation, as well as further described health benefits of MTX in neurodegenerative diseases, suggest that MTXs might be valuable to treat or prevent AD but should be included in a healthy diet in general.

## 4. Materials and Methods

### 4.1. Chemicals, Reagents and Standards

Theobromine, pentoxifylline, propentofylline, HPLC-grade pyridine, phenyl isothiocyanate (PITC) and ammonium acetate were purchased from Merck (Darmstadt, Germany), whereas caffeine, theophylline and all other chemicals including high performance liquid chromatography (HPLC)-grade water, ethanol, and methanol were purchased from Fisher Scientific (Schwerte, Germany). The following standards from Avanti Polar Lipids were used for normalization: 06:0 PC (DHPC), 19:0 Lyso PC, 06:0 SM (d18:1/6:0), Cer d18:1/6:0, D7-cholesterol and Splash^®^ II Lipidomix^®^ Mass Spec Internal Standard.

### 4.2. Cell Culture and MTX Treatment

SH-SY5Y and N2a cells were cultivated at 37 °C and 5% CO_2_ in Dulbecco´s modified Eagle´s medium (DMEM) containing the cell specific supplements listed in Table 1.

Sixteen hours prior to incubation, the FBS content in DMEM was reduced to 1% to elucidate the potential effect of xanthine derivatives on lipids being also present in FBS. The different cell lines were long-term incubated with a concentration of 100 µM MTX or with HPLC-grade H_2_O as a solvent control every 24 h for six days.

To check whether the used conditions are suitable to monitor a potential effect of methylxanthines on the different cell lines, we monitored the gene expression of genes known to be influenced by methylxanthines [29]. Indeed, in line with literature, we found several genes to be influenced by methylxanthines (see Supplemental Figure S9), showing that the used conditions were in principle appropriate to study the effect of methylxanthines.

### 4.3. Cytotoxicity Measurement

Cytotoxicity was measured utilizing Lactate Dehydrogenase Cytotoxicity Assay Kit (Cayman Chemical, Ann Arbor, MI, USA) according to the manufacturer’s protocol; 100 µM of the different methylxanthines did not show any major effects on cell viability (<3%) (see Supplemental Figure S10). As several other studies were performed with 100 µM methylxanthines, this concentration was chosen to achieve comparable conditions. In respect to the timeline, an incubation of six days was preferred, because this longer methylxanthine exposition allows adaption of the cellular lipid homeostasis, in particular lipids with a longer half-life, to these compounds.

### 4.4. Sample Preparation

Cells were washed two times with ice-cold HPLC-grade water before harvesting and mechanical homogenization in 180 µL water via Minilys (Peqlab, Erlangen, Germany) for 60 s on maximum intensity. Bicinchoninic acid assay according to Smith et al. (1985) was used to measure the protein content of the homogenized samples [78]. Homogenates were adjusted to a protein amount of 10 mg/mL in HPLC-grade water.

### 4.5. Lipid Extraction

For detection of phospholipids and TAGs, lipids were extracted using solid/liquid lipid extraction method as described in detail in Lauer et al. (2021) [79]. Into the wells of a 96-well filter plate (0.45 µM; Merck), circles of Whatman blotting paper with a diameter of 6 mm were placed, and the plate was fixed on a 96-deep well plate (Fisher Scientific). A standard mixture and 10 µL of each prepared sample were added to the Whatman papers and samples were dried under a nitrogen flow (1–2 bar) for 45 min. Afterwards, 20 µL of 5% PITC (*v*/*v*) diluted in ethanol/water/pyridine (1:1:1, *v*/*v*/*v*) were added to the samples and incubated for 20 min at room temperature. After drying the samples for 45 min under nitrogen, the lipids were extracted using 300 µL 4.93 mM ammonium acetate in methanol and shaking the plate for 30 min at 450 rpm on a plate shaker (IKA, Staufen, Germany). By centrifugation for 2 min at 500× *g*, the liquid samples were transferred into the 96-deep well plate. Before covering the plate with a silicone mat, the samples were diluted with 600 µL 5 mM ammonium acetate in methanol/water (97:3, *v*/*v*). The plate was further shaken for 2 min at 450 rpm at room temperature, and afterwards mass spectrometry analysis was performed.

Extraction of cholesterol was performed according to Sandhoff et al. (1999) [80] with modifications. In brief, 10 µL of each prepared sample were mixed with cholesterol standard and 100 µL 1,4-dioxane, and sonicated for 10–20 s at room temperature. Afterwards, samples were dried using a vacuum concentrator (Speedvac, Thermo Fisher Scientific, Waltham, MA, USA), solved in 50 µL 1,4-dioxane, and sonicated up to six times for 30 s. After a centrifugation for 20 min at 20,800× *g* and room temperature, the supernatant was transferred into a new tube and samples were dried. Samples were solved in 20 µL of a 31.4 mM sulfurtrioxid-pyridine solution by sonication for 10 s, and afterwards they were incubated for 30 min at room temperature. Sulfidation was stopped by adding 2.1 µL 314.1 mM bariumacetate solution and incubation for 10 min at room temperature and 60 min at 4 °C. After dilution with 120 µL methanol and centrifugation at 20,800× *g* and room temperature for 10 min, the supernatant was transferred into a 96-deep well plate. Then, 600 µL methanol per well was added and the plate was covered with a silicone mat. The plate was shaken for 2 min at 450 rpm and room temperature before mass spectrometry analysis was performed.

The extraction method for phospholipids and TAGs is based on [79,81], and in this context, reliability, recovery rate and linearity were checked previously: the averaged extraction efficiency was >80.7% with an intra-day variance of 3.9%, and the linearity of this extraction method was R^2^ > 0.96, as published previously in [79,81].

### 4.6. Mass Spectrometry

For the detection of different lipid species, including phospholipids, TAG and cholesterol, a 4000-quadropole linear-ion trap (QTrap) equipped with a Turbo Spray ion source (AB SCIEX, Darmstadt, Germany) was used. Measurements were performed in triplicates using the Analyst 1.4.2 software (AB SCIEX, Darmstadt, Germany) with help of an autosampler of the Agilent HPLC 1200 for direct injection. We used this direct infusion with a high degree of automatization in our flow injection analysis to avoid difficulties with concentration alterations and chromatographic abnormalities to ensure robustness. Beside these advantages, a potential caveat should also be emphasized: in principle, isobaric masses between different lipid classes cannot be distinguished. Therefore, we focused on multiple reaction monitoring (MRM) transitions, which show no overlay or only an overlay to very minor and slightly existing species, which are presented in the supplementary material (Supplemental Table S2). Specificity of the detected lipids with the same headgroup (PC and SM species) was checked using the LIPID MAPS website (https://www.lipidmaps.org/, accessed on 26 August 2021). Only MRM species known from literature to fulfill this property, and which have been extensively used in other shotgun lipidomics approaches [82,83,84,85,86,87,88,89,90], were chosen. The mass transitions, which were chosen for the MRM, are listed in Supplementary Table S3.

Moreover, by not using a HPLC-based method, matrix effects could potentially occur. To evaluate whether matrix effects might influence the outcome of our study, we measured the matrix effects by adding external standards in known ratios. Further experiments revealed that these ratios between the external standards were not altered in samples treated with methylxanthines compared to samples treated with solvent control. Potential matrix effects were at maximum 1.12 % and on average 1.05 %.

Besides the technical triplicates, at least five independent biological replicates per cell line and methylxanthine were analyzed (see Supplemental Table S1). For the lipid analysis which was performed in the scan type MRM with a measurement period of three minutes, the parameters listed in Table 2 were used.

### 4.7. Statistical Analysis

Counts per second for each MRM pair were extracted via the Analyst 1.4.2 software (AB Sciex, Darmstadt, Germany). For every measured lipid, we normalized it to its respective lipid class standard. Following the normalization process, the mean per technical triplicate was formed for each ratio. Statistical analysis was carried out with R (R Core Team 2020; Vienna, Austria; https://www.R-project.org/, accessed on 26 August 2021). P value calculation for each parameter was carried out using Dunnett‘s test to compare each MTX against the control group. Volcano plots were created via the R package “EnhancedVolcano “ (Kevin Blighe, Sharmila Rana and Myles Lewis (2020); version 1.6.0.; https://github.com/kevinblighe/EnhancedVolcano). Images were created using Inkscape (Inkscape Project (2020); Inkscape; retrieved from https://inkscape.org, accessed on 26 August 2021). Error bar graphs represent the standard error of the mean. Significance was set at * *p*  ≤  0.05, ** *p*  ≤  0.01 and *** *p*  ≤  0.001. An overview of the lipid changes mediated by the analyzed methylxanthines in SH-SY5Y and N2a cells is given in Supplemental Table S4.

## Figures and Tables

**Table 1:** Composition of the different cultivation media for the used cell lines. FBS: fetal bovine serum (GE Healthcare Life Sciences, Chalfont St. Giles, UK). NEAA: non-essential amino acids. Pen-Strep: Penicillin/Streptomycin.

Cell Line	FBS	NEAA	Pen-Strep	Sodium-Pyruvate	L-Glutamine
SH-SY5Y	10%	0.1 mM	/	/	/
N2a	10%	0.1 mM	1%	1 mM	2 mM

**Table 2:** Parameters of mass spectrometry used for the detection of different lipid species.

Parameter	Ceramide	Cholesterol	PC, TAG
Curtain Gas (CUR)	10 psi	10 psi	20 psi
Temperature (TEM)	200 °C	0 °C	200 °C
Ion Source Gas 1 (GS1)	40 psi	19 psi	40 psi
Ion Source Gas 2 (GS2)	50 psi	0 psi	50 psi
Interface Heater (ihe)	on	on	on
Collisionally activated dissociation gas (CAD)	medium	medium	medium
Ion Spray Voltage (IS)	4500 V	−4500 V	5500 V
Entrance Potential (EP)	10 V	−10 V	10 V
Collision Cell Exit Potential (CXP)	14 V	−5 V	15 V

## Data Availability

Not applicable.

## Supplementary Materials

The following are available online at https://www.mdpi.com/article/10.3390/ijms23042295/s1.
