# Supplementary material for: Methylxanthines Induce a Change in the AD/Neurodegeneration-Linked Lipid Profile in Neuroblastoma Cells
Source: Int J Mol Sci. 2022 Feb 18;23(4):2295. doi: 10.3390/ijms23042295 (PMC8875332; doi:10.3390/ijms23042295)
Supplement: Supplementary file 1 [file ijms-23-02295-s001.zip › ijms-1376935_Supplementary Material_revised version.pdf]

Supplementary Material

Mass spectrometry analysis of lipid changes induced by methylxanthines in different cell lines

Daniel Janitschke [1,†], Anna Andrea Lauer [1,†], Cornel Manuel Bachmann [1], Jakob Winkler [1], Lea Victoria Griebsch [1], Sabrina Melanie Pilz [1], Elena Leoni Theiss [1], Heike Sabine Grimm [1], Tobias Hartmann [1,2] and Marcus Otto Walter Grimm [1,2,3,*]

Suppplemental Figure S1

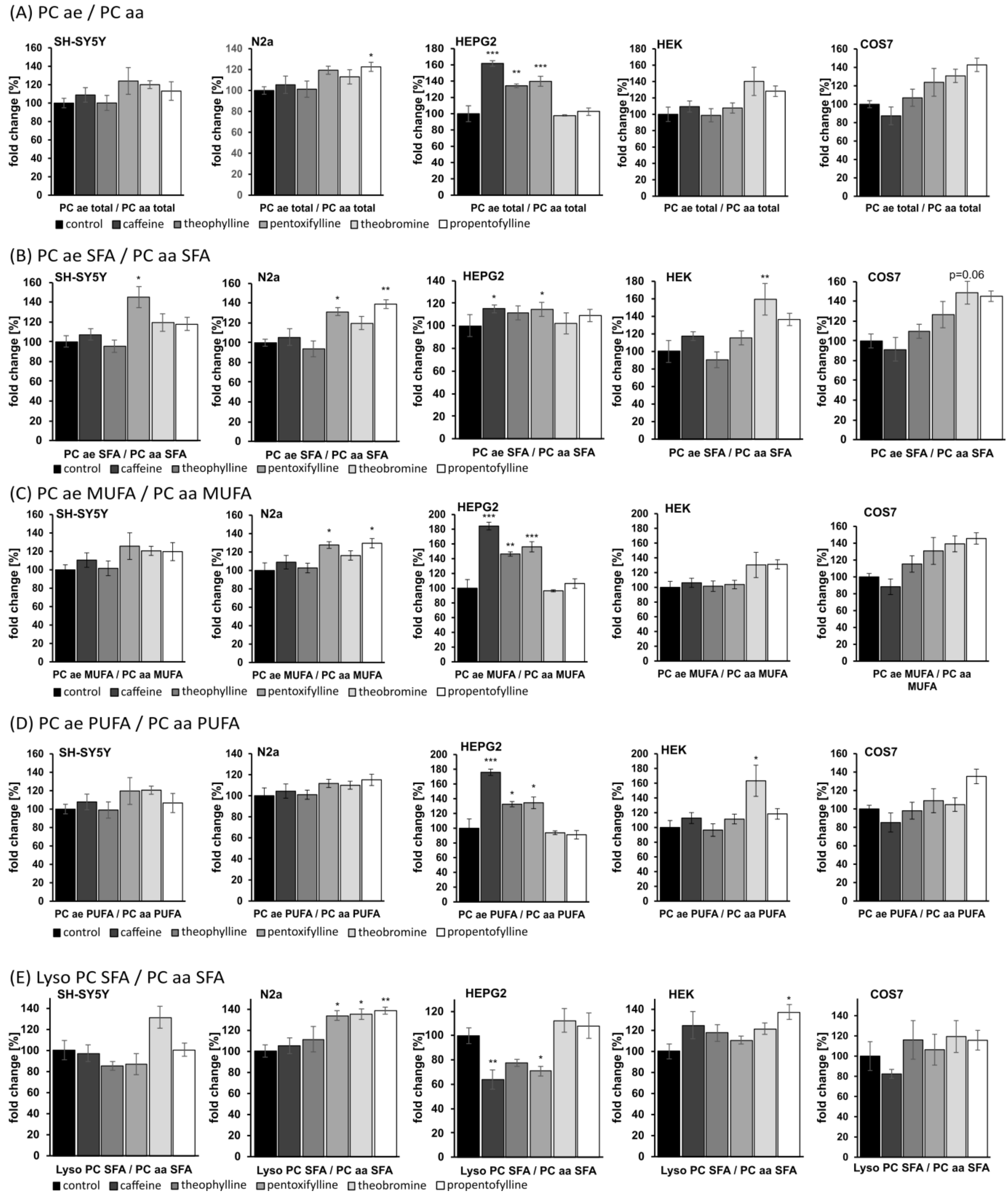

Suppplemental Figure S1 (continued)

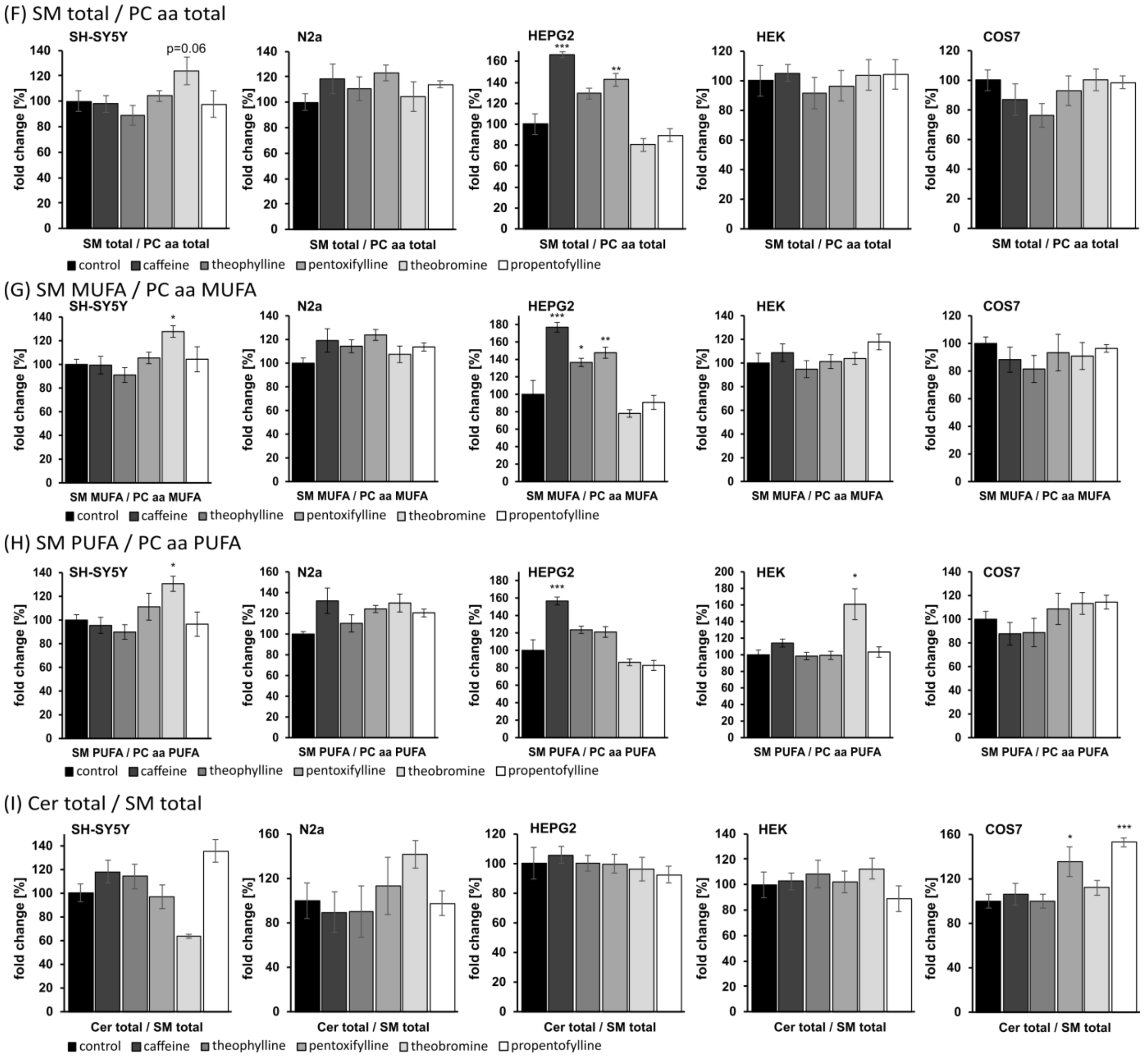

Supplementary Figure S1: Effect of different methylxanthines (caffeine, theophylline, pentoxifylline, theobromine, and propentofylline) on PCae / PCaa, Lyso PC/PCaa, SM/PCaa, and Cer/SM ratios in SH-SY5Y-, N2a-, HEPG2-, HEK-, and COS7 cells. Error bars represent the standard error of the mean (SEM) and statistical significance was set as * p ≤ 0.05, ** p ≤ 0.01 and *** p ≤ 0.001.

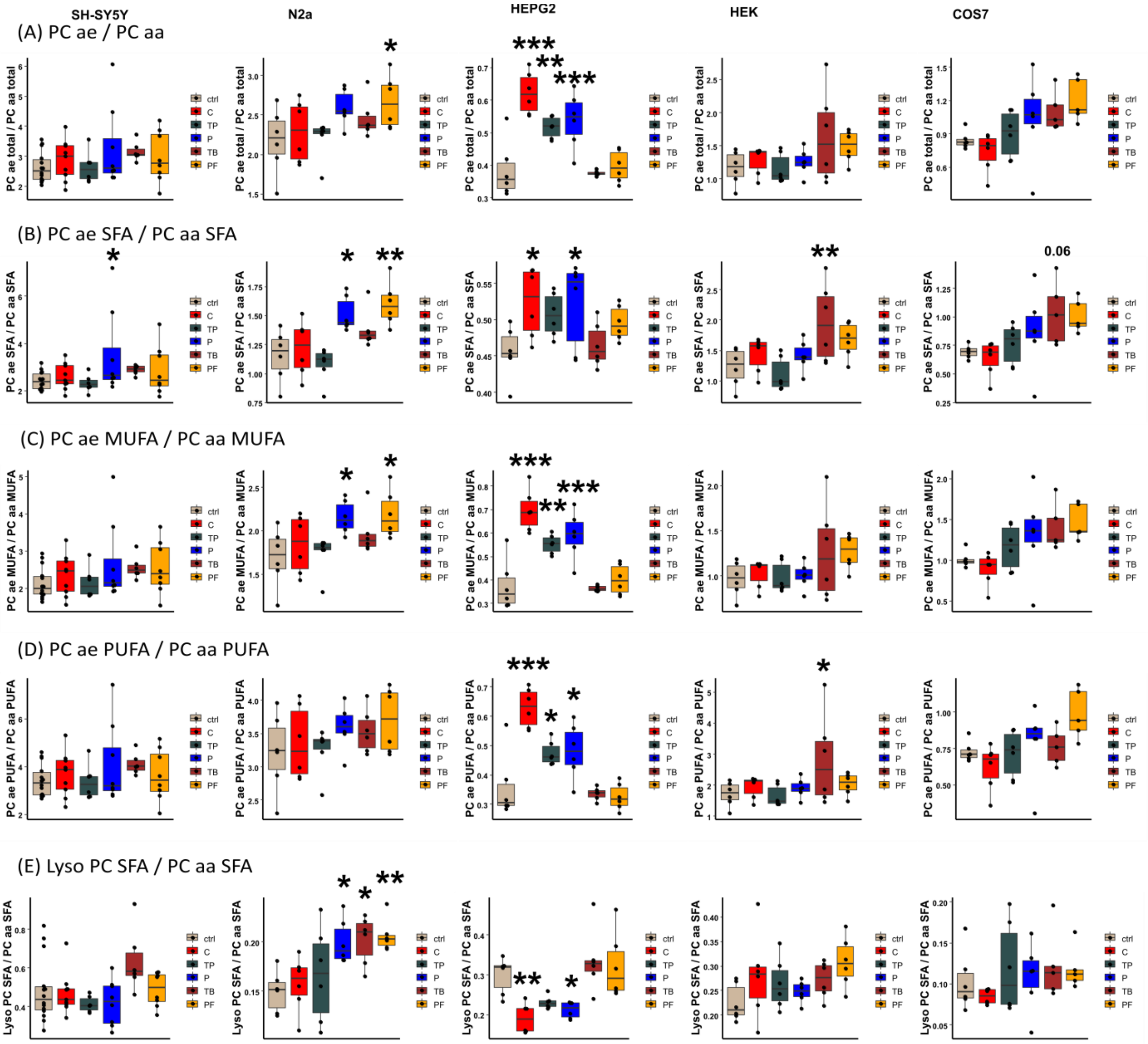
SH-SY5Y
N2a
HEPG2
HEK
COS7
(A) PC ae / PC aa
PC ae total / PC aa total
(B) PC ae SFA / PC aa SFA
PC ae SFA / PC aa SFA
0.06
(C) PC ae MUFA / PC aa MUFA
PC ae MUFA / PC aa MUFA
(D) PC ae PUFA / PC aa PUFA
PC ae PUFA / PC aa PUFA
(E) Lyso PC SFA / PC aa SFA
Lyso PC SFA / PC aa SFA
ctrl
C
TP
P
TB
PF

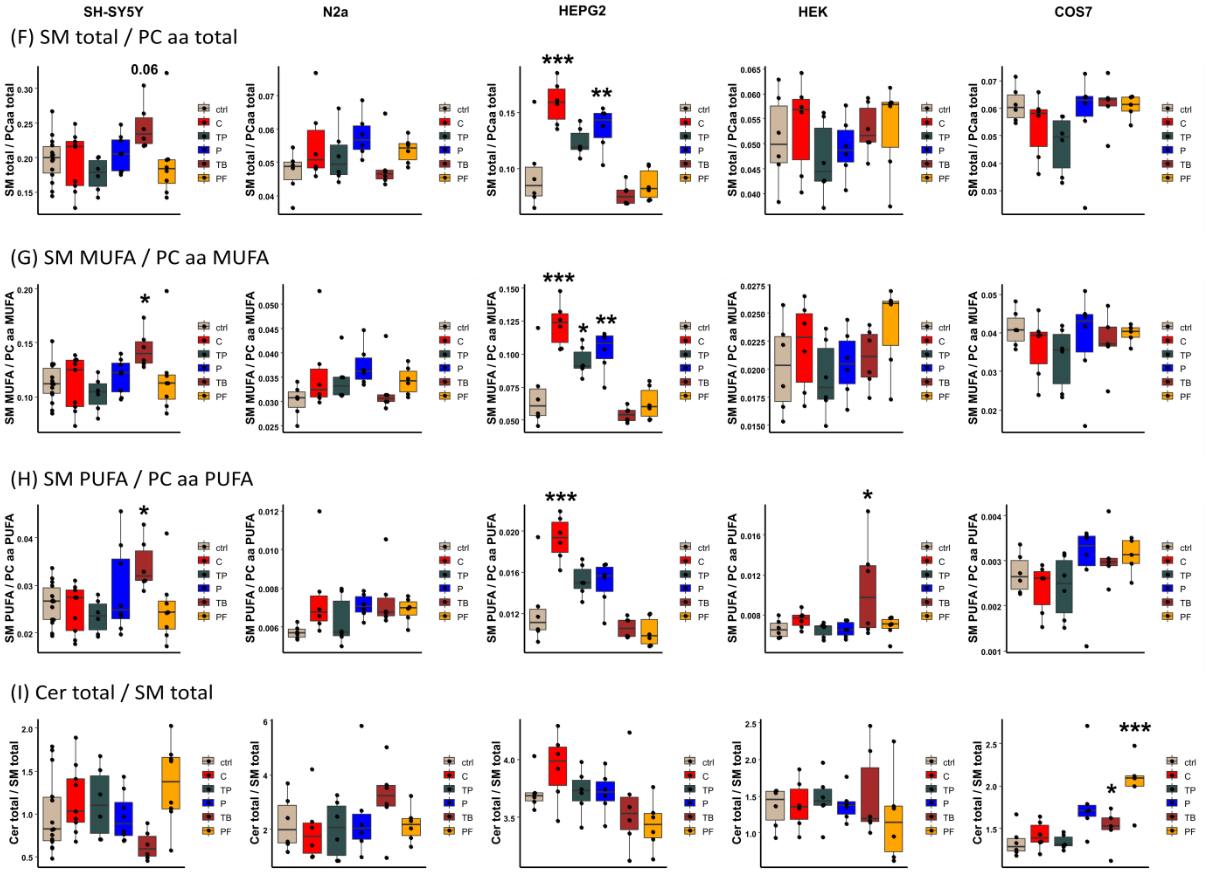

Supplementary Figure S2: Effect of different methylxanthines (caffeine, theophylline, pentoxifylline, theobromine, and propentofylline) on PCae / PCaa, Lyso PC/PCaa, SM/PCaa, and Cer/SM ratios in SH-SY5Y-, N2a-, HEPG2-, HEK-, and COS7 cells shown as boxplots. Error bars represent the standard error of the mean (SEM) and statistical significance was set as * p ≤ 0.05, ** p ≤ 0.01 and *** p ≤ 0.001.

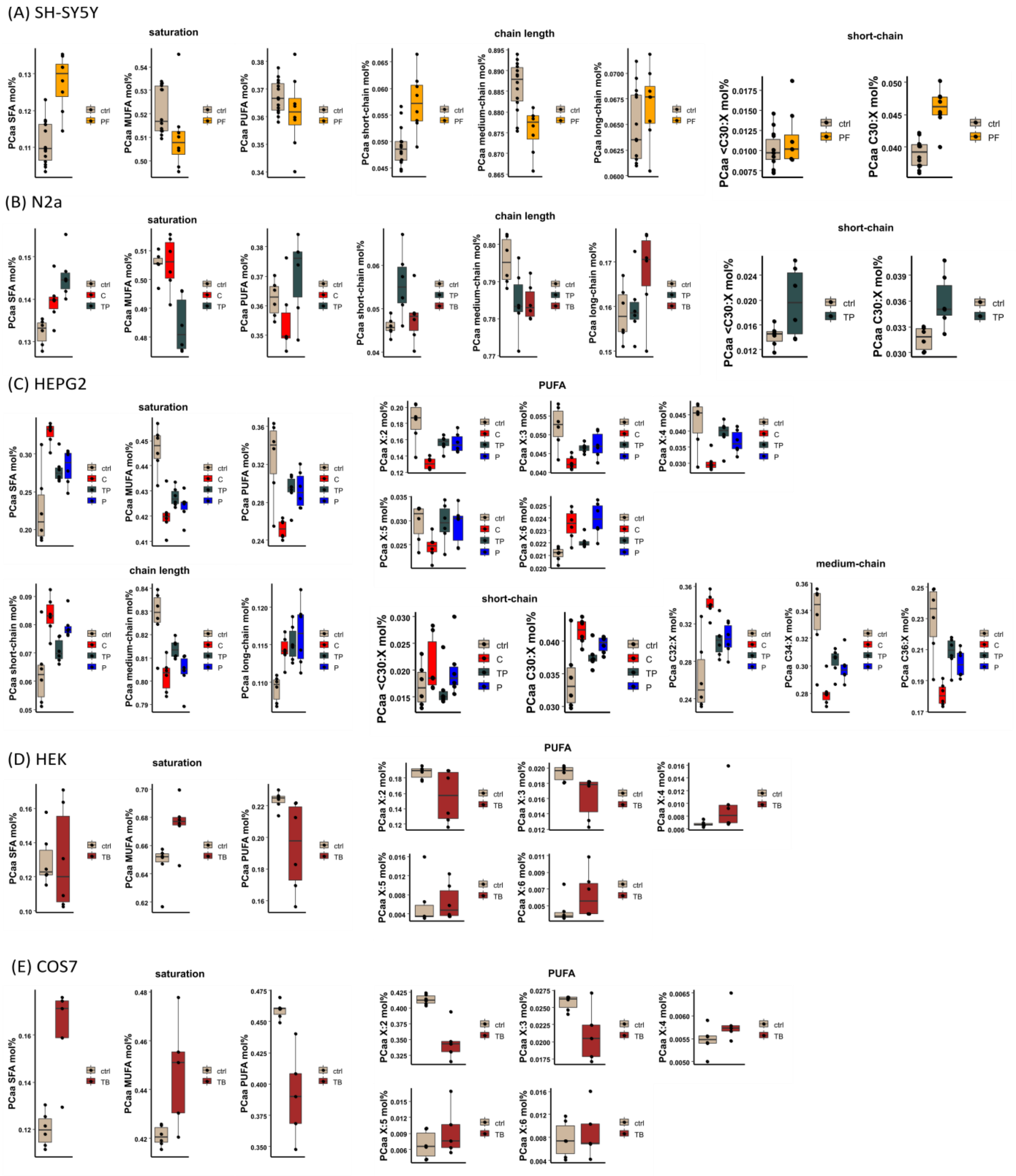

Supplementary Figure S3: Alterations of phosphatidylcholine (PCaa) levels in different cell lines (SH-SY5Y, N2a, HepG2, HEK and COS7) in presence of the analyzed MTXs caffeine (C), theophylline (TP), pentoxifylline (P), theobromine (TB) and propentofylline (PPF) shown as boxplots (in accordance to Figure 2).

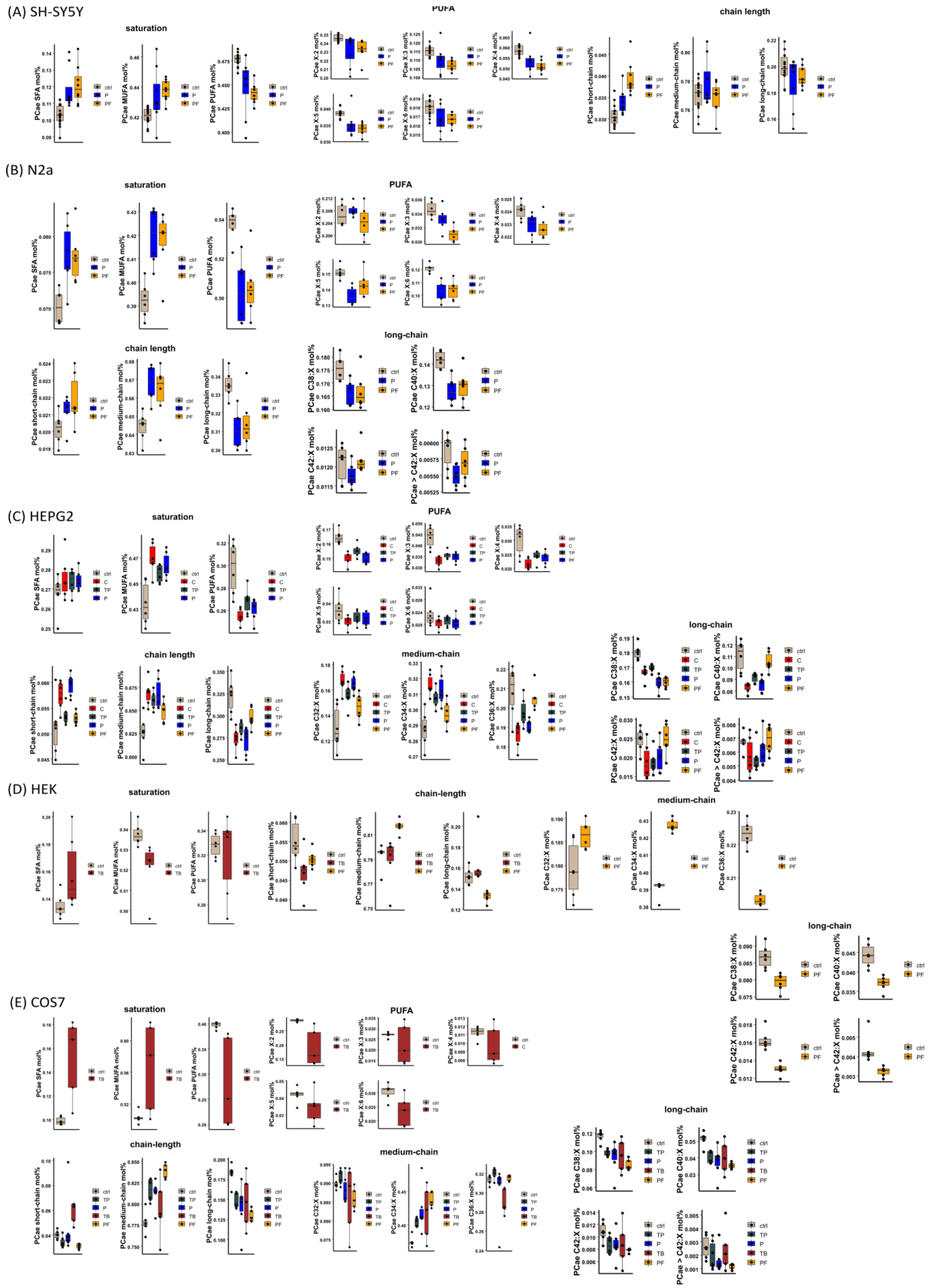

Supplementary Figure S4: Alterations of phosphatidylcholine plasmalogens (PCae) levels in different cell lines (SH-SY5Y, N2a, HepG2, HEK and COS7) in presence of the analyzed MTXs caffeine (C), theophylline (TP), pentoxifylline (P), theobromine (TB) and propentofylline (PPF) shown as boxplots (in accordance to Figure 3).

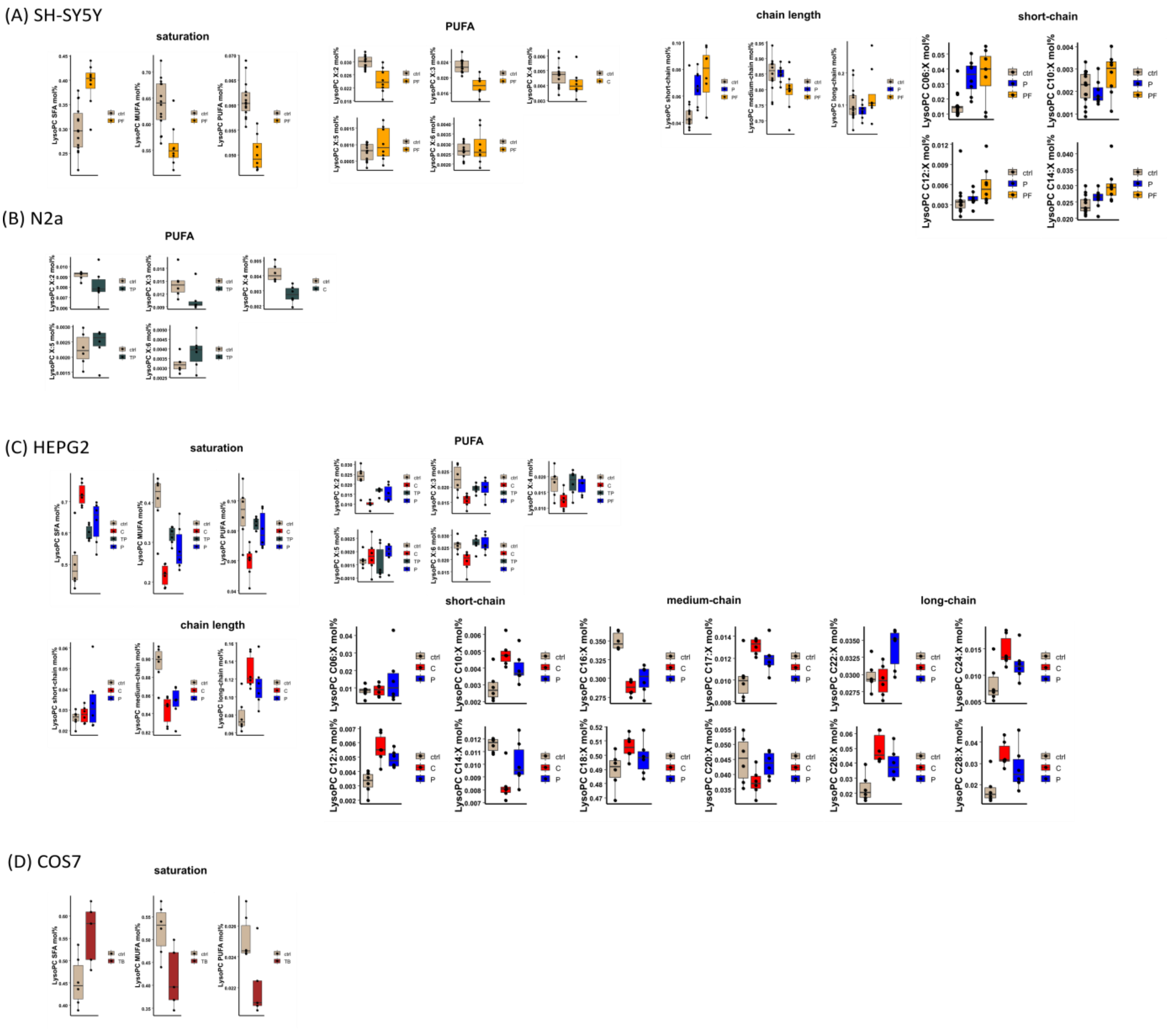

Supplementary Figure S5: Alterations of lyso-phosphatidylcholine (lyso-PC) levels in different cell lines (SH-SY5Y, N2a, HepG2, HEK and COS7) in presence of the analyzed MTXs caffeine (C), theophylline (TP), pentoxifylline (P), theobromine (TB) and propentofylline (PPF) shown as boxplots (in accordance to Figure 4).

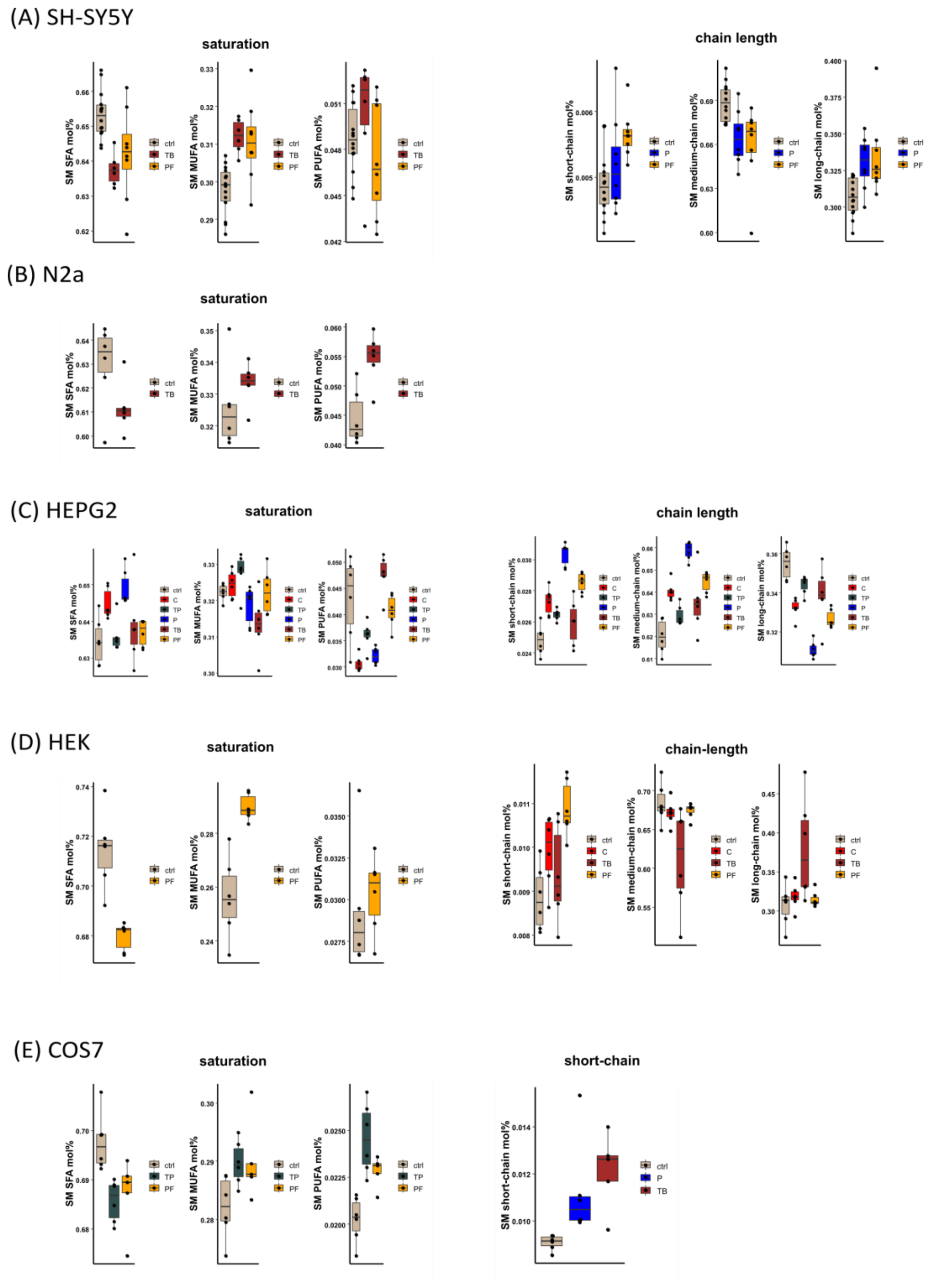

Supplementary Figure S6: Alterations of sphingomyeline (SM) levels in different cell lines (SH-SY5Y, N2a, HepG2, HEK and COS7) in presence of the analyzed MTXs caffeine (C), theophylline (TP), pentoxifylline (P), theobromine (TB) and propentofylline (PPF) shown as boxplots (in accordance to Figure 5).

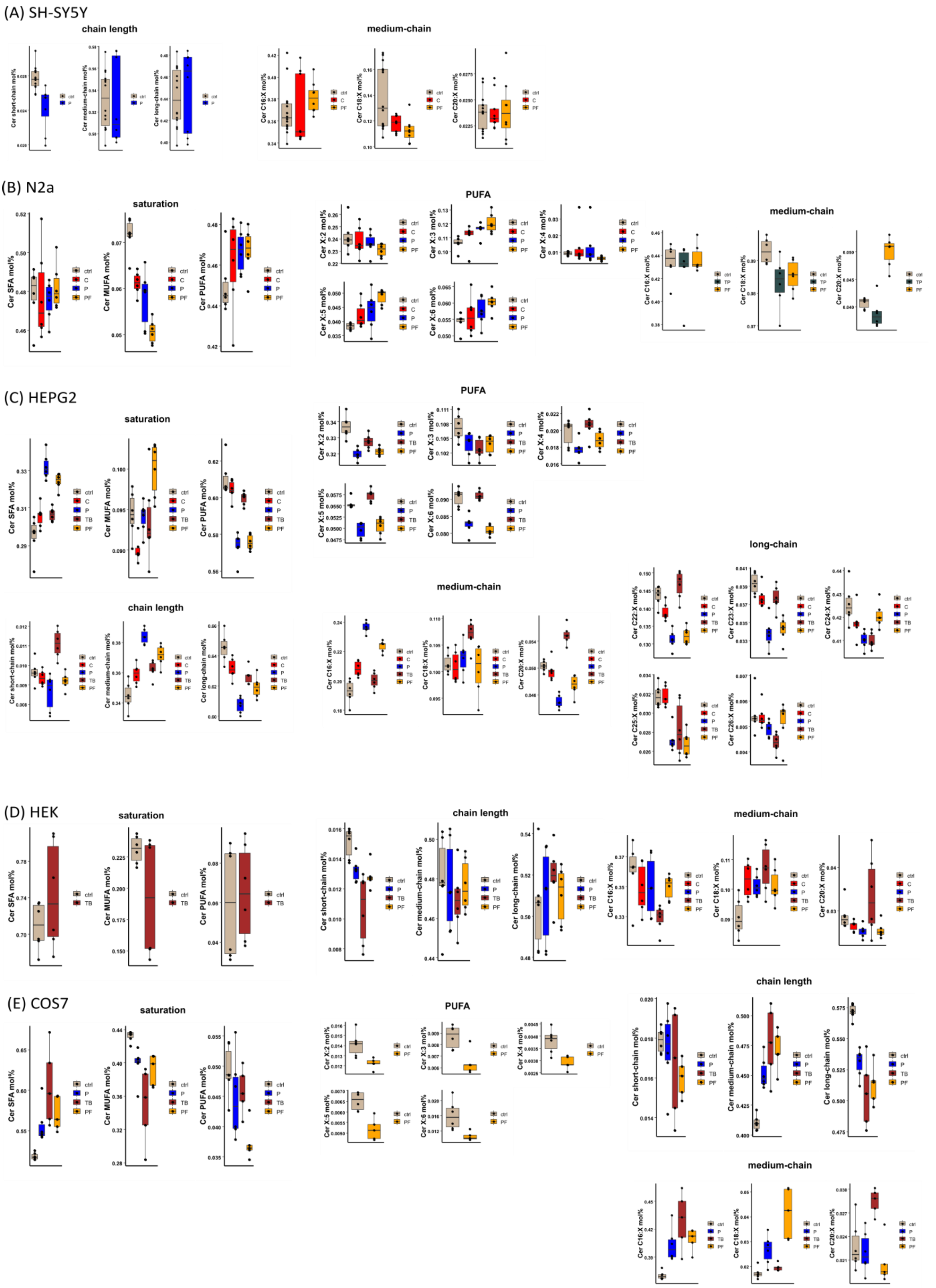

Supplementary Figure S7: Alterations of ceramide (Cer) levels in different cell lines (SH-SY5Y, N2a, HepG2, HEK and COS7) in presence of the analyzed MTXs caffeine (C), theophylline (TP), pentoxifylline (P), theobromine (TB) and propentofylline (PPF) shown as boxplots (in accordance to Figure 6).

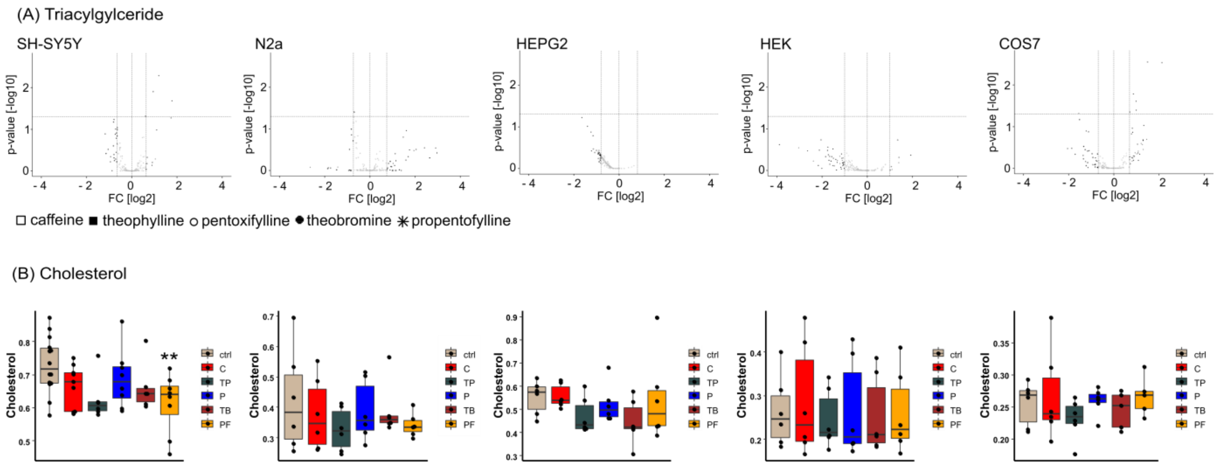

Supplementary Figure S8: Effect of different methylxanthines (caffeine, theophylline, pentoxifylline, theobromine, and propentofylline) on TAG and cholesterol levels in SH-SY5Y-, N2a-, HEPG2-, HEK-, and COS7 cells shown as volcano plots and boxplots. Error bars represent the standard error of the mean (SEM) and statistical significance was set as * p ≤ 0.05, ** p ≤ 0.01 and *** p ≤ 0.001.

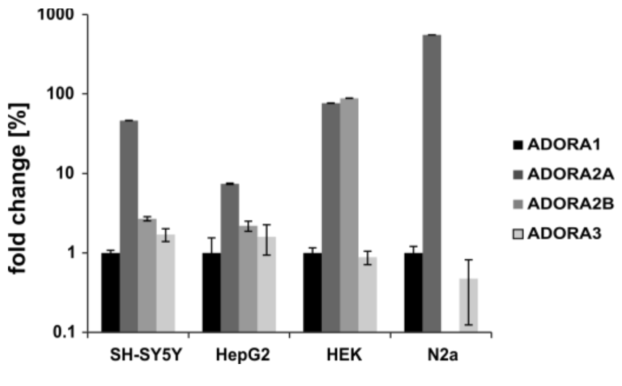

Supplementary Figure S9: Gene expression pattern of ADORA1, ADORA2A, ADORA2B and ADORA3 in SH-SY5Y, HepG2, HEK and N2a cells analyzed by qPCR measurement. Error bars represent the standard error of the mean (SEM).

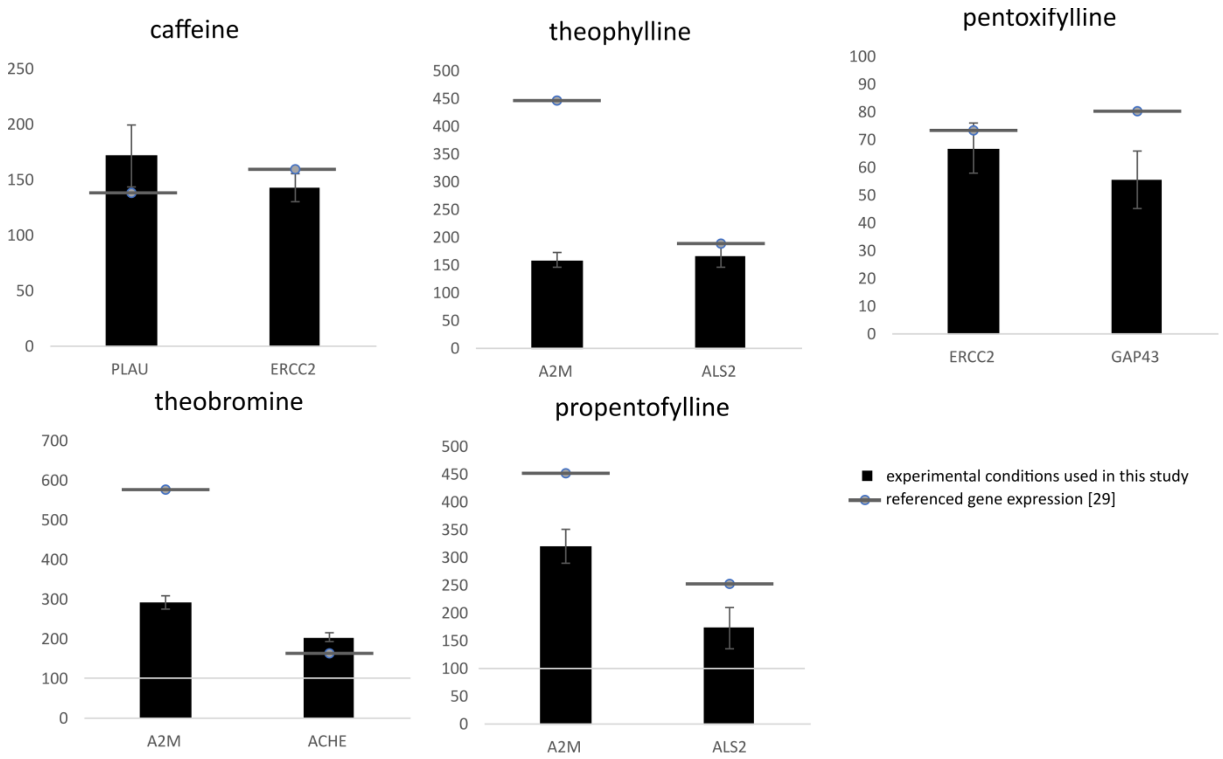

Supplementary Figure S10: Verification of the biological effects of the methylxanthines on known alterations in gene expressions (as reported in [29]) compared to the effects on gene expression under the experimental conditions used in this study. Error bars represent the standard error of the mean (SEM).

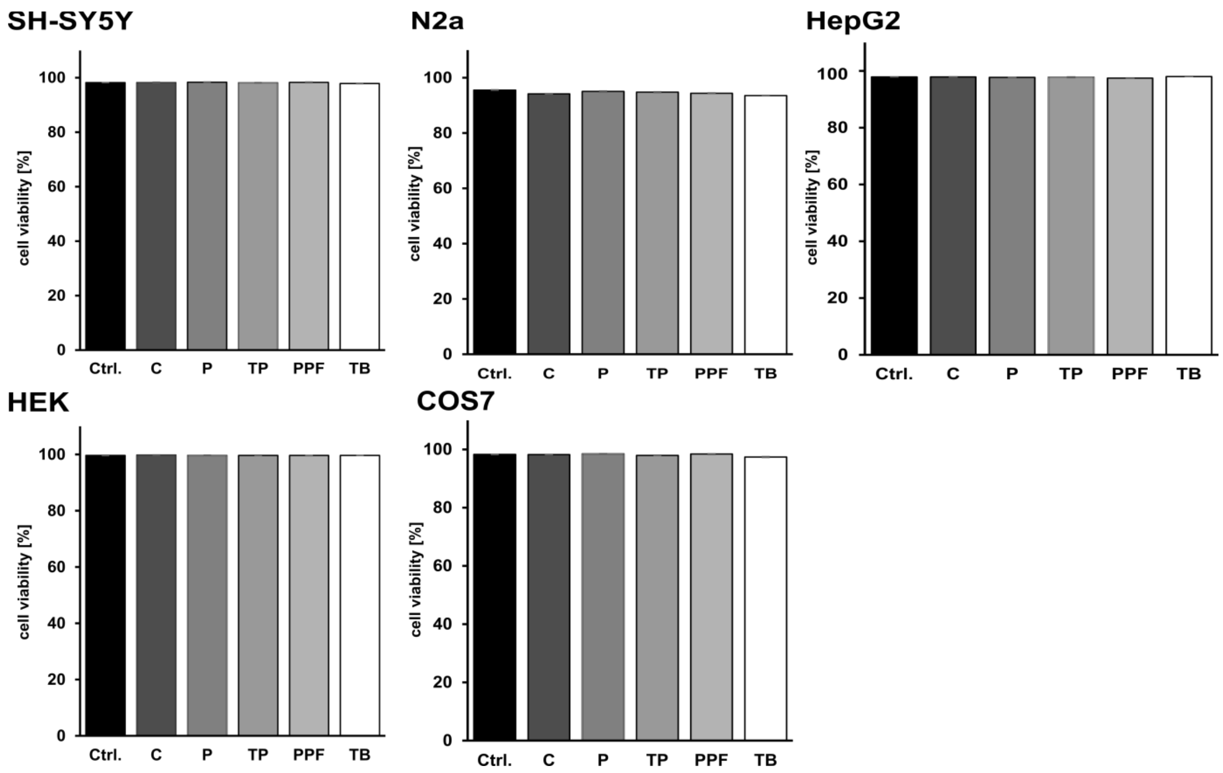

Supplementary Figure S11: Cell viability after treatment with different methylxanthines or solvent control in SH-SY5Y, N2a, HepG2, HEK and COS7 cells. Error bars represent the standard error of the mean (SEM).

Supplemental Table 1. Number of biological replicates for each analyzed cell line and each incubated methylxanthine.

Cell line	Methylxanthine	Biological replicates
**SH-SY5Y**	Solvent control	n=14
	Caffeine	n=9
	Theophylline	n=6
	Pentoxifylline	n=8
	Theobromine	n=6
	Propentofylline	n=8
**N2a**	Solvent control	n=6
	Caffeine	n=6
	Theophylline	n=6
	Pentoxifylline	n=6
	Theobromine	n=6
	Propentofylline	n=6
**HEK**	Solvent control	n=6
	Caffeine	n=6
	Theophylline	n=6
	Pentoxifylline	n=6
	Theobromine	n=6
	Propentofylline	n=6
**HepG2**	Solvent control	n=6
	Caffeine	n=6
	Theophylline	n=6
	Pentoxifylline	n=6
	Theobromine	n=6
	Propentofylline	n=6
**COS7**	Solvent control	n=6
	Caffeine	n=6

	Theophylline	n=6
	Pentoxifylline	n=6
	Theobromine	n=5
	Propentofylline	n=5
